# Numerical Study on Fluid Flow Behavior and Heat Transfer Performance of Porous Media Manufactured by a Space Holder Method

**DOI:** 10.3390/ma17112695

**Published:** 2024-06-03

**Authors:** Xianke Lu, Yuyuan Zhao, Yue Zhang, Mian Wu

**Affiliations:** 1 School of Mechanical and Automotive Engineering, Ningbo University of Technology, Ningbo 315211, China; xianke@nbut.edu.cn (X.L.); wumian91@nbut.edu.cn (M.W.); 2 School of Engineering, University of Liverpool, Liverpool L69 3GH, UK; 3 China Merchants Chongqing Communications Research & Design Institute Co., Ltd., Chongqing 400067, China; zhangyue8@cmhk.com

**Keywords:** open-cell metal foam, porous flow, pressure drop, heat transfer coefficient, forced convection, CFD

## Abstract

The velocity field and temperature field are crucial for metal foams to be used as a heat exchanger, but they are difficult to obtain through physical experiments. In this work, the fluid flow behavior and heat transfer performance in open-cell metal foam were numerically studied. Porous 3D models with different porosities (55–75%) and pore sizes (250 μm, 550 μm, and 1000 μm) were created based on the porous structure manufactured by the Lost Carbonate Sintering method. A wide flow velocity range from 0.0001 m/s to 0.3 m/s, covering both laminar and turbulent flow regimes, is fully studied for the first time. Pressure drop, heat transfer coefficient, permeability, form drag coefficient, temperature and velocity distributions were calculated. The calculated results agree well with our previous experimental results, indicating that the model works well. The results showed that pressure drop increased with decreasing porosity and increasing pore size. Permeability increased and the form drag coefficient decreased with increasing porosity, and both increased with increasing pore size. The heat transfer coefficient increased with increasing velocity and porosity, whereas it slightly decreased with increasing pore size. The results also showed that at high velocity, only the metal foam close to the heat source contributes to heat dissipation.

## 1. Introduction

Thermal management has always been an important issue in the electronic industry since an overly high temperature will not only reduce the performance of electronic components, but also shorten their service life [1,2]. With the development of electric vehicles, thermal management becomes more important because there is a recommended operating temperature for the battery and this temperature is related to the performance and safety of the vehicles [3,4]. Traditional designs, such as louvered fins and slit fins, are hitting bottlenecks due to their limits in mass and specific surface area. Open-cell metal foams as an alternative for the traditional fins are attracting more and more attentions [5,6,7].

An open-cell metal foam is a cellular structure consisting of a network of metal ligaments (copper and aluminum) and fluid-filled (usually water and air) pores. The ratio of pore volume to the total volume is named porosity with a value normally ranging from 50% to 95%. The metal network provides a low density, good thermal conductivity, and an excellent fluid mixing function. The specific surface area of metal foam reaches 800 cm^2^/g, and combined with the good fluid permeability, it provides favorable conditions for heat convection [8]. These properties make open-cell metal foams widely used in the field of heat transfer, chemical catalysis and filtration [9].

Open-cell metal foams can be manufactured by investment casting, powder metallurgy sintering, and vapor deposition process methods [10,11]. Open-cell copper foam manufactured by the Lost Carbonate Sintering (LCS) process which is based on the powder metallurgy sintering technique is a promising type of material for use as a heat exchanger [12]. These foams have two grades of pores that provide more surface area for heat transfer than other cast foams. In our previous research, the heat transfer performance and fluid flow behavior in these LCS foams were experimentally investigated by the pressure drop method and flow visualization method [13,14]. The effects of flow rate and flow regime on pressure drop and heat transfer were systematically analyzed, which were generally consistent with other cellular materials. However, some challenges are difficult to overcome in physical experiments, such as low flow control, low pressure, and low velocity measurements and temperature distribution throughout the metal foam. Numerical simulation methods are powerful tools for studying coupled flow and heat transfer in metal foam, providing more information than physical experiments [15,16,17].

Model construction is the most essential and important step in numerical simulation. Metal foams are complex cellular structures and it is impossible to precisely describe their structure characteristics. In addition, it is unnecessary to use a complex model when we focus on the fundamentals. Therefore, a simplified structure analogous to the metal foam is preferred in research. Lu et al. [18] studied the effects of foam density, pore size, and inlet length on the temperature distribution by using simple cubic unit cells with a constant wall temperature setup and a discrepancy was found due to oversimplification of the transport equation. Boomsma et al. [19] defined an idealized open-call model using eight 14-sided tetrakaidecahedron cells (Kelvin model) and used periodic boundary conditions to model an infinity matrix. They found the simulated pressure drop values were 25% lower than the experimental values due to the lack of wall effects. Kopanidis et al. [20] built a solid ligament foam model based on a volume composed of six tetrakaidecahedra and two irregular dodecahedra (W-P model). This model results in a 0.3% reduction in surface energy compared to the Kelvin model used by Boomsma. Results showed that the heat transfer and pressure drop significantly depend on entrance effects, near-wall effects and heat conduction. Recently, Hu et al. [21] used the Kelvin model to study the forced convection of porous metal used as a heat exchanger in a turbine engine at high velocity (>20 m/s) which is difficult to achieve in experiment. A quadratic relationship between pressure drop and velocity, and a logarithmic relationship between the volumetric heat transfer coefficient and velocity were confirmed at high velocity. Yang et al. [22] investigated the interstitial heat transfer coefficient of high-porosity foam using the W-P model under rotational conditions. A relationship consistent with the experimental results was established between the heat transfer coefficient and the structural parameters, and it was also found that the interstitial heat transfer coefficient increases with increasing flow rate and rotation speed. Gauna and Zhao [23] studied pressure drop and heat transfer in porous media but did not consider the effect of natural convection on heat transfer.

However, the difference between the currently existing models and the LCS structure is so great that the current model cannot be used directly. In this study, a geometry model is proposed to imitate the LCS porous structure. The design process of the model, combined with the fabrication process of the LCS metal foam, is fully described. The pressure drop and heat transfer coefficient are systematically investigated for models with different porosities, pore sizes and flow rates. Temperature distribution and velocity distribution are analyzed at various parameters. This study can provide more information for the structure design of metal foams used as heat exchangers.

## 2. Numerical Model and Method

### 2.1. Model Construction

**Figure 1:**
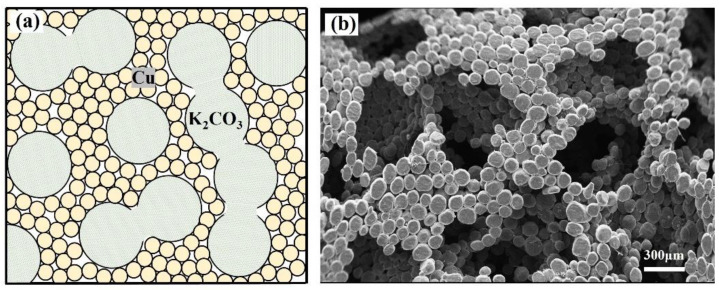
Structure of porous copper sample manufactured by a space holder method: (**a**) schematic diagram of the mixture of pore agent and metal powder and (**b**) scanning electron micrograph of sintered sample.

The present model is based on an open-cell metal foam manufactured by the space holder method [11]. In manufacturing, large carbonate particles and fine metal powder are mixed, compacted and sintered. Carbonate particles are removed by a dissolution or decomposition process, leaving an open-cell metal foam. The pore distribution and connectivity of the pores are random on a macroscopic scale, but regularity can be found at the microscopic scale. A schematic drawing of the microstructure and scanning electron micrograph of copper foam sample manufactured by this method are shown in Figure 1. The large pores are the replica of the carbonate particles and these pores connect to each other through a small hole which is formed by the tangent point. The pore wall is composed of sintered copper particles. The porosity of the metal foam manufactured by this process ranges from 50% to 80%. The median porosity of such foam makes the Kelvin model [24] infeasible as it can only show the structure characteristics of the high-porosity foam. The face-centered cubic (FCC) packed unit cell has a packing factor of 74%, which matches the current porosity very well. All models in the present study are constructed based on the FCC unit cell.

### 2.2. Physical Model

**Figure 2:**
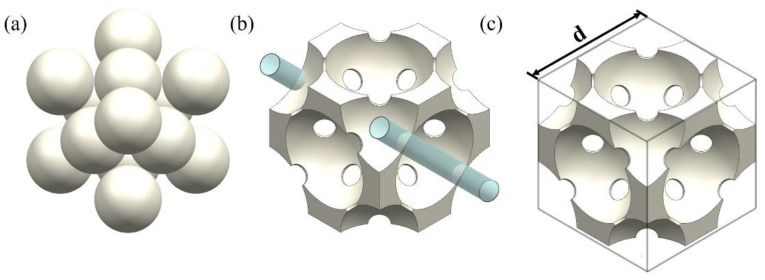
The design of the REV unit cell. From (**a**) FCC packing to (**b**) negative replicas and then to (**c**) final unit cell.

**Figure 3:**
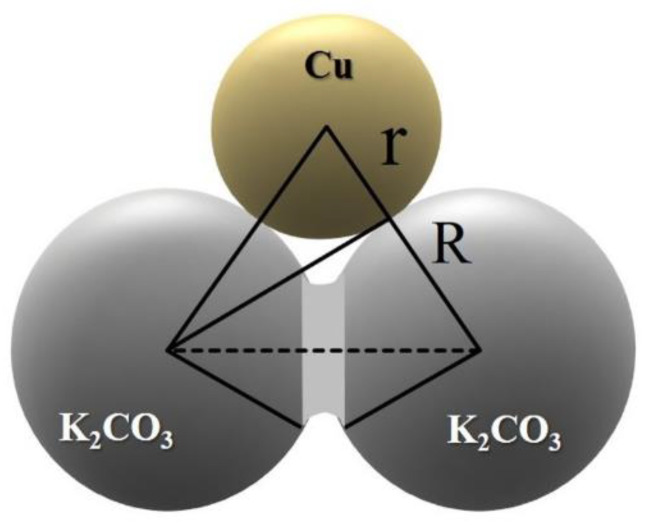
Schematic drawing showing the geometric relationship of the cylindrical hole and neighboring particles.

The representative elementary volume (REV) is used to represent the porous structure of metal foam. Figure 2 shows the design procedure of the REV unit cell. The unit cell of the REV is the negative replica of the FCC unit cell in which the spherical pores are located where the atoms are in the FCC unit cell. The pores are connected through cylindrical holes at the tangent point. The porosity of the REV is controlled by varying the distance of the neighboring pores and the diameter of the cylindrical hole. Figure 3 shows the geometric relationship of the cylindrical hole and neighboring pore and particles. The radius of the cylindrical hole, *r_c_*, reflects the connectivity of the neighboring pores and can be determined by the pore radius, *R*, and metal particle radius, *r*. The distance between pores can be calculated by Equation (1) [23]:

(1) $$l_{p}=2R\left(\sqrt[{3}]{\frac{\pi }{(3\sqrt{2})\epsilon }}-1\right)$$

The radius of the cylindrical hole is calculated by Equation (2) [25]:

(2) $$r_{c}=\sqrt{\frac{\omega \cdot A_{SC}}{12\pi }}$$

where *ω* is the coordination number, representing the number of connected pores with its neighbors. The coordination number is 12 for the FCC, but for the foam manufactured by a space holder method, the value is much lower and can be estimated by Equation (3) [25]:

(3) $$\omega =\frac{2}{\left(1-\sqrt{\frac{\phi +2}{\sqrt{\phi^{2}+6\phi +5}}}\right)\left(1-\phi +\frac{\phi }{\epsilon }\right)}$$

where *ϕ* is radius ratio between the pore and metal particle. *A_SC_* is the area of the sphere crown and can be calculated by Equation (4) [25]:

(4) $$A_{SC}=2\pi R^{2}\left(-\frac{\phi +2}{\sqrt{\phi^{2}+6\phi +5}}\right)$$

**Figure 4:**
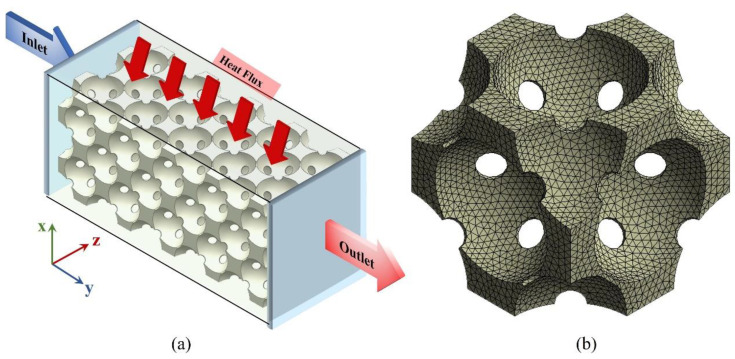
Computational domain and boundary conditions of the (**a**) geometry and (**b**) grid of the unit cell.

Models with pore sizes of 250 μm, 550 μm and 1000 μm and porosities of 55%, 60%, 65%, 70% and 75% were built. In this study, the diameter of the cylindrical hole was calculated assuming that the diameter of the metal particle is 50 μm. The details of all the physical models are listed in Table 1. Figure 4 shows the typical geometry model and boundary conditions of simulation. Table 2 lists details of the boundary condition. According to the previous study, the result is unchanged when the number of unit cells in the mean flow direction is equal to or larger than four [23]. Therefore, five cells were built in the mean flow direction. The thickness (in heat flow direction) of the sample significantly affects the heat transfer performance of the metal foam. To investigate the effect of thickness, models with 1–4 layers at porosities of 55% and 75% and a pore size of 550 μm were built. For other results, models with 3 layers were used. The dimensions of a typical three-layered model are around 4.5 × 0.9 × 2.7 mm^3^. A total of 21 models were built and more than 400 calculations were performed.

### 2.3. Governing Equation and Boundary Condition

Heat transfer and fluid flow were simulated by ANSYS Fluent [26]. The working fluid velocity ranges from 0.0001 to 0.3 m/s, which covers both laminar and turbulent. A heating block was placed on the top of the model so that the natural convection within the porous model was avoided. The highest temperature of the heating block and the working fluid is less than 100 °C. Radiation is negligible and the thermo-physical properties such as thermal conductivity are temperature independent [27]. In this study, water was used as the working fluid and it was assumed to be incompressible. The temperature field of the porous model is calculated by solving the conjugate heat transfer problem. In the case of the porous model, conjugate heat transfer refers to the simultaneous heat transfer through both the solid material of the porous medium and the fluid flowing through the pores. The conjugate heat transfer problem is typically solved using numerical methods such as finite element analysis or computational fluid dynamics. These methods allow for the simulation of heat transfer within complex geometries and can account for different modes of heat transfer such as conduction, convection, and radiation. The temperature field in the porous medium is influenced by the heat transfer between the solid matrix and the fluid, as well as by any heat sources or sinks present in the system. Solving the conjugate heat transfer problem is crucial for applications such as heat exchangers, thermal insulation or energy storage systems where efficient heat transfer is essential.

The incompressible working fluid is governed by the Navier–Stokes equations, which includes the continuity, momentum and energy equation given by Equations (5)–(7).

(5) $$\mathrm{Continuity}\;\frac{\partial }{\partial x_{i}}\rho u_{i}=0$$

(6) $$\mathrm{Momentum}\;\frac{\partial }{\partial x_{i}}\left(\rho u_{i}u_{j}\right)=\frac{\partial P}{\partial x_{j}}+\frac{\partial }{\partial x_{i}}\left(\mu \frac{\partial u_{j}}{\partial x_{i}}\right)$$

(7) $$\mathrm{Energy}\;\frac{\partial }{\partial x_{i}}\left(\rho u_{i}C_{p}T\right)=\frac{\partial }{\partial x_{i}}\left(k\frac{\partial T}{\partial x_{i}}\right)$$

where *ρ* is the fluid density, *u* is the velocity, *P* is the pressure, *μ* is the fluid viscosity, *T* is the temperature and *k* is the thermal conductivity.

For laminar flow, the laminar model was used, while for turbulent flow, the standard *k-ξ* model was used. The critical velocity for the transition from laminar flow to turbulent flow was chosen according to both the experimental and simulation results. Our previous experiments [14] on porous copper reveal that the transition velocity is around 0.03 m/s. Simulation results shows that when the inlet velocity is higher than 0.04 m/s, the laminar model cannot make the simulation converge. According to the ANSYS documentation, if a simulation is set up using laminar flow, but the real flow is turbulent, convergence is difficult and the result is not correct. In the present study, simulation results agree well with the experimental results and therefore the transition velocity is around 0.04 m/s.

As shown in Figure 4, the computational domain is composed of an inlet region, porous model and outlet region. The inlet region was left for long enough that the flow was fully developed before entering the porous model [13]. A constant temperature of 300 K was set in the inlet boundary; a constant zero pressure was set in the outlet boundary; a constant heat flux of 250 KW/m^2^ was set on the top of the heating block; and the interface between the metal and fluid was set as a non-slip boundary condition and the other two sides of the domain were set as symmetric. In this case, the heat is transferred from the heating block to the metal foam through conduction and then to the working fluid through convection. The temperature and velocity fields in the fluid domain were calculated and the temperature field in the solid domain was calculated.

### 2.4. Mesh and Solution

The 3D model was constructed using Unigraphics NX (UG) software (https://plm.sw.siemens.com/en-US/nx/) and then imported into the Integrated Computer Engineering and Manufacturing (ICEM) software (https://www.ansys.com/training-center/course-catalog/fluids/introduction-to-ansys-icem-cfd) to generate mesh. Different meshing methods and accuracies were applied in different regions to save calculating time. Coarse mesh was generated in the inlet, outlet and heating block region and fine mesh was generated in the porous model region. We also conducted a mesh number dependence study, and the results showed that when the number of meshes was higher than 6 million, the simulation results became stable. Considering accuracy and efficiency, approximately 10 million meshes were used in a single model. The overall quality of the mesh was higher than 0.85. The results were considered to be converged when the residuals of the variables were lowered by six orders of magnitude. The raw data were exported to files and then imported into MATLAB (https://www.mathworks.com/products/matlab.html) for post-processing.

### 2.5. Pressure Drop

It is difficult to choose two points in a porous medium to directly calculate the pressure drop due to the large difference in pressure in different regions of the porous medium. Therefore, the pressure drop was calculated from the pressure outside the porous model. When calculating the pressure drop, two planes were selected. One is located just in front of the inlet section and the other is located just after the outlet section. The average pressure on the two planes was calculated separately and the pressure drop value is equal to the pressure difference between the two planes. The same measurement method was used in our previous experimental study, and the consistency of the method facilitates data comparison.

### 2.6. Heat Transfer Coefficient

The overall heat transfer coefficient of the porous model was calculated according to the Newton’s cooling law given by Equation (8)

(8) $$J=h\left(T_{c}-T_{in}\right)$$

where *J* is the input heat flux, *h* is the heat transfer coefficient, *T_c_* is the average temperature of the contact region between heating block and porous media and *T_in_* is the temperature of the water in the inlet region.

## 3. Results and Discussion

### 3.1. Pressure Drop

**Figure 5:**
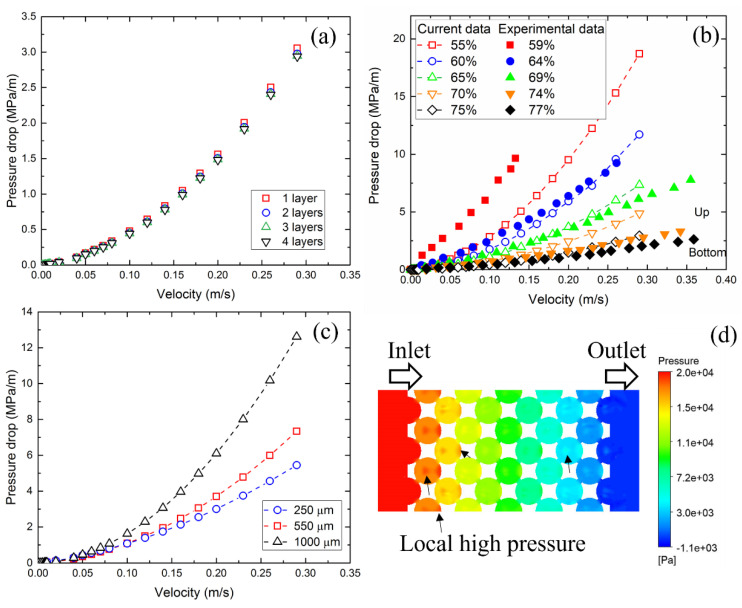
(**a**) Effect of the number of layers on pressure drop; (**b**) variation in pressure drop with velocity for different models with pore size of 550 μm and comparison with experimental data [28]; (**c**) pressure drop comparison of models with porosity of 65% and different pore sizes; and (**d**) pressure field in a model with porosity 65%, pore size 550 μm and velocity 0.2 m/s.

Figure 5 shows the variation in pressure drop with velocity for models with different layers, porosities and pore sizes. To verify the effect of the number of layers (FCC unit cell) on the pressure drop, models with 1–4 layers were built, and the pressure drop at different velocities was calculated for each model. Figure 5a shows that the pressure drop is slightly higher for the one-layer model and the other models have the same pressure drop. Therefore, the three-layer model was used for all simulations. Figure 5b shows that the pressure drop increases dramatically with velocity and a typical pressure drop–velocity curve was achieved, which is consistent with previous physical experiments [28]. The pressure drop for the experimental sample with a porosity of 59% is higher than that for the model with a porosity of 55%. This is because the pore connectivity of the copper foam sintered by the space holder method is poor, while the pore connectivity of the model is not affected by the porosity. It also shows that the rate at which the pressure drop increases with velocity is much higher for the low-porosity model than for the high-porosity model.

The relationship between pressure drop and velocity depends on the flow regime. At low velocity, viscous force dominates the flow and a linear relationship can be observed. This linear relationship can be described by Darcy’s law [29]: $\frac{∆P}{L}=\frac{\mu V}{K}$, where Δ*P* is pressure drop, *L* is the length of the porous media, *V* is the Darcian velocity which equals to the volume flow rate divided by the cross-sectional area of the flow channel and *K* is the permeability of the porous medium. The flow regime that applies Darcy’s law is called the Darcy regime. Usually, the Darcy regime is very narrow compared to other regimes.

At high velocity, the inertial force plays a significant role and dominates the flow. The Forchheimer equation is commonly used to describe the relationship between pressure drop and velocity. The equation is in the form of:

(9) $$\frac{∆P}{L}=\frac{\mu }{K}V+\rho CV^{2}$$

where *C* is the form drag coefficient. The Forchheimer equation is a quadratic function and applies the non-Darcy flow regime. Fitting curves based on quadratic function are plotted in the Figure 5b and a high degree of consistency is observed.

Figure 5b,c show that both porosity and pore size have a significant effect on the pressure drop. For the model with a pore size of 550 μm, the pressure drop for porosity 55% is nearly eight times that of porosity 75% at 0.25 m/s. For a specific porosity and velocity, e.g., 65% and 0.25 m/s, the pressure drop for pore size 1000 μm is 2–3 times that of pore size 250 μm. For a specific porosity, a large pore size means a low number of pores, resulting in high tortuosity, while a small pore size means a high number of pores, resulting in low tortuosity. The tortuosity has a great influence on the pressure drop. For porous media with high tortuosity, the fluid has to travel a longer distance from point A to point B, and the pressure drop will be higher [30].

### 3.2. Permeability and Friction Factor

**Figure 6:**
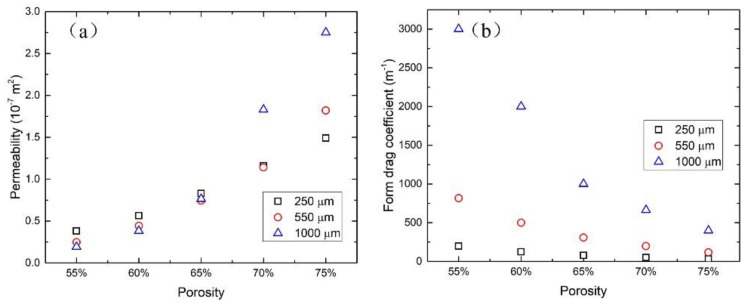
The variation of (**a**) permeability and (**b**) form drag coefficient with porosity and pore size.

Permeability and the inertial drag coefficient were calculated by comparing the fitting equation with the Forchheimer equation. The values of permeability (K) and the inertial drag coefficient are listed in Table 3. The variation in permeability and the inertial drag coefficient with porosity and pore size are shown in Figure 6. As porosity increases, the permeability increases and the form drag coefficient decreases. The effect of pore size on permeability and the form drag coefficient is related to porosity. The effect of pore size on permeability is much stronger at high porosity than at low porosity. For the form drag coefficient, the opposite is true.

The flow regimes cannot be identified from the pressure drop–velocity figure but can be figured out from the relationship between friction factor and velocity. The friction factor is defined as:

(10) $$f=\frac{∆P}{L}\cdot \frac{D}{\rho V^{2}}$$

**Figure 7:**
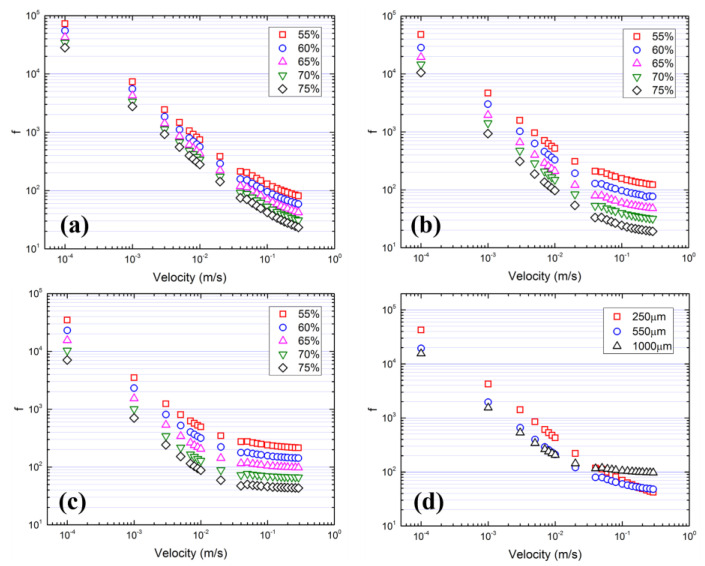
The friction factor. (**a**) 250 μm, (**b**) 550 μm, (**c**) 1000 μm and (**d**) porosity is 65%.

Figure 7 shows the variation in friction factor with velocity. The linear relationship between friction factor and velocity at low velocity corresponds to the laminar flow regime. A constant friction value will be reached at high velocity, which corresponds to the turbulent flow regime. A transition range exists between laminar and turbulent flow regimes. Figure 7 shows that high porosity and a large pore size lead to low friction because of weak impedance of the solid matrix and a low surface area. A large pore size also makes the flow become stable turbulent faster.

### 3.3. Temperature Distribution

#### 3.3.1. Effect of Flow Rate

**Figure 8:**
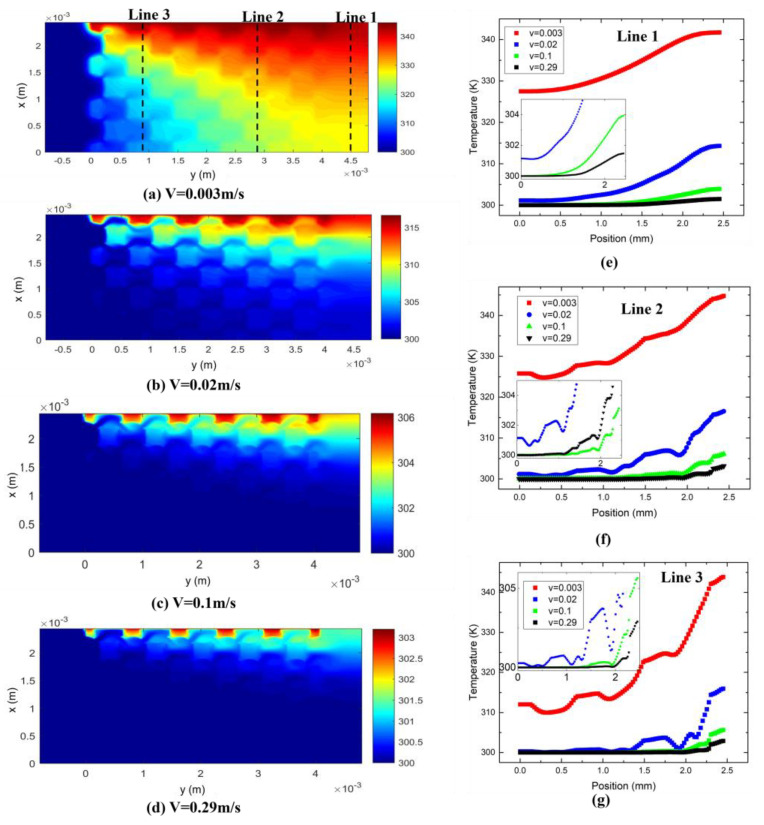
(**a**–**d**) Temperature distribution in metal foam under different flow rates. Heat source is on the top of the wall. Temperature distribution along the (**e**) line 1, (**f**) line 2 and (**g**) line 3 in (**a**–**d**).

Figure 8 shows the effect of flow rate on the temperature distribution in metal foam with a pore size of 550 μm and a porosity of 65%. At different velocities, the difference in the highest temperature in the metal foam can reach up to 40 K. For example, the highest temperature is more than 340 K at a velocity of 0.003 m/s, while it is only about 300 K at a velocity of 0.29 m/s. The uniformity of temperature distribution throughout the whole metal foam also varies greatly at different velocities. When the velocity is less than 0.02 m/s, the heat reaches the bottom part (away from the heat source) of the metal foam, while when the velocity is higher than 0.02 m/s, the bottom is not heated at all, which means that at high velocity, the bottom of the metal foam contributes nearly nothing to heat transfer because the heat has been taken away before it gets that far.

Figure 8e–g show the temperature distribution along the three reference lines in Figure 8a. Line 2 and line 3 are located on the metal foam, while line 1 is located in the outlet area. Figure 8f,g show that the temperature in the metal foam is higher than that of the fluid even though they are in direct contact. This is more pronounced in the area near the inlet because heat conduction in the metal foam happens faster than convection between the metal foam and the fluid.

#### 3.3.2. Effect of Porosity

**Figure 9:**
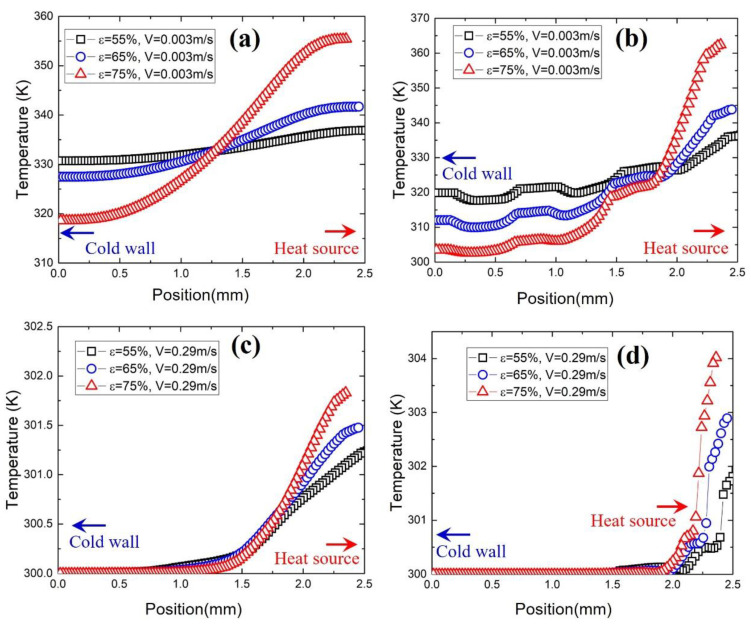
Temperature distribution along line 1 and 3 for samples with different porosities at different flow rates. The pore size of all samples is 550 μm. (**a**,**c**) show the temperature along line 1 and (**b**,**d**) show the temperature along line 3.

Figure 9 shows the temperature distribution along line 1 and 3 for samples with a porosity of 55%, 65% and 75% at low and high flow rates. Porosity has a great impact on the temperature distribution and this effect is more pronounced at a low flow rate near the inlet (line 3) and outlet (line 1). For example, in Figure 9b, the temperature difference between the heat source and cold wall is 60 K for the sample with a porosity of 75%, while the difference is only 15 K for the sample with a porosity of 55%. This is because, at low velocity (v = 0.003 m/s), the fluid cannot effectively take heat away. Therefore, if the heat cannot be transferred quickly to the cold part on the metal foam, there will be a large temperature difference between the heat source part and the cold wall part, which is the case for the high-porosity sample. However, if the heat can be efficiently transferred to the other side of sample due to high thermal conductivity, the temperature will be more uniform, which is the case for the low-porosity sample.

At high velocity (v = 0.29 m/s), the effect of porosity on the temperature distribution is also affected by the velocity, especially near the cold wall part. This is because the fast-moving fluid takes heat away before it is transferred to the cold wall part. Therefore, for samples with different porosities, the temperature near the cold wall part is the same. In addition, due to the strong influence of the high flow rate, the temperature difference in the heat source part of different samples is only 1–2 K.

#### 3.3.3. Effect of Pore Size

**Figure 10:**
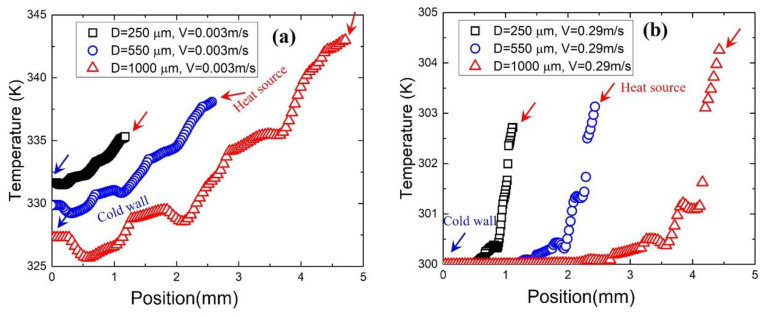
Temperature distribution along line 2 for metal foam with porosity of 55% and pore sizes of 250 μm, 550 μm and 1000 μm at velocity of (**a**) 0.003 m/s and (**b**) 0.29 m/s.

Although all models have the same number of cell layers in the Z direction, a different pore size means different unit cell dimensions and different model heights. Figure 10 shows the effect of pore size on the temperature distribution along line 2 for metal foam with different pore sizes. In regions close to the heat source, the larger the pore size, the higher the temperature for all velocities. However, in the cold wall part, the smaller the pore size, the higher the temperature at low velocity, and the same temperature at high velocity. It has been proved that the thermal conductivity of metal foam depends insignificantly on pore size [31,32,33], so the temperature difference on the metal foam is mainly caused by the height difference of the model.

### 3.4. Velocity Distribution

#### 3.4.1. Effect of Flow Rate

**Figure 11:**
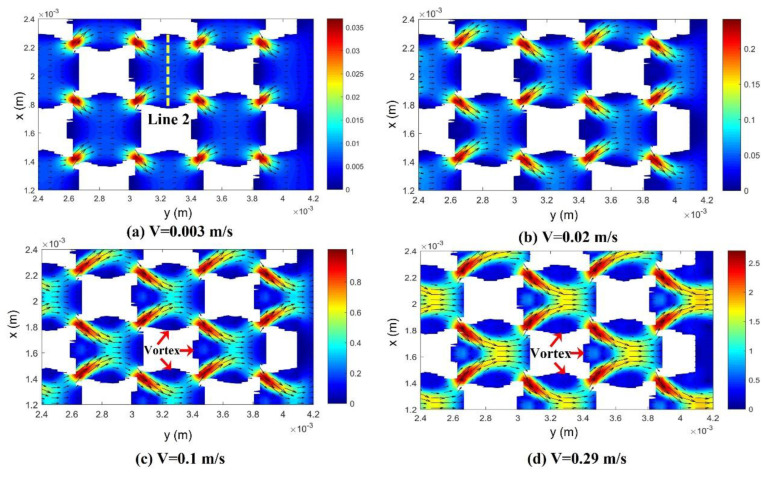
Pore-scale velocity distribution at different flow rates. Pore size: 550 μm; porosity: 65%.

Figure 11 shows the x–y plane of the pore-scale velocity distribution of a sample with a pore size of 550 μm and a porosity of 65% at different flow rates. When the flow velocity is less than 0.02 m/s, the direction of the mean velocity at the pore scale is inlet to outlet, while when the flow velocity is great than 0.1 m/s, vortices can be found in the top, bottom and left part of a pore. As discussed in the Figure 8, a fast-moving (v > 0.1 m/s) fluid has efficiently removed the heat from the metal matrix, making the lower part of the metal foam approximately the same temperature as water. This greatly enhanced convective heat transfer capacity is mainly attributed to pore-scale vortices. The high temperature near the heat source region proves that heat conduction through the metal foam is still faster than heat convection.

**Figure 12:**
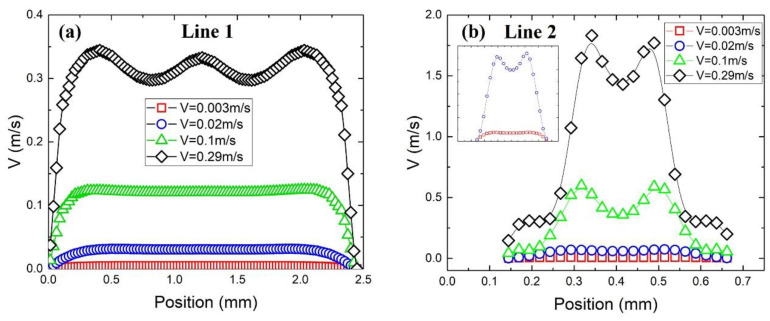
Velocity profiles along a reference line at different flow velocities for the sample with pore size of 550 μm and porosity of 65%. (**a**) Line 1 is located at y = 4.8 × 10^−3^ m and (**b**) line 2 is located at the position marked in Figure 11a.

Figure 12 shows the velocity profiles along a reference line at different flow rates for the sample with a pore size of 550 μm and a porosity of 65%. Line 1 is located at the outlet region without the metal foam. When the velocity is less than or equal to 0.1 m/s, the velocity profile in the non-near-wall region is quite flat. This is due to the good mixing function of metal foam. Meanwhile, when the velocity reaches 0.29 m/s, a ‘‘W’’-shaped profile is observed in the non-near-wall region. This demonstrates that a longer distance after the outlet is required at high flow rates to obtain a uniformly distributed velocity. Line 2 is located at the position marked in Figure 11a and its velocity profile is shown in Figure 12b. A typical “M” shape is observed due to the merging of two tributaries in the pore. However, due to the influence of eddy flow, the velocity near the pore wall when the flow rate is 0.29 m/s is higher than that near the pore wall under other flow conditions. 

#### 3.4.2. Effect of Porosity and Pore Size

Porosity and pore size mainly affect the transition velocity (critical Reynolds number) from laminar flow to turbulent flow. Their effect on velocity distribution is insignificant. This has been proved in our previous physical experiments [13,14].

### 3.5. Heat Transfer Coefficient

**Figure 13:**
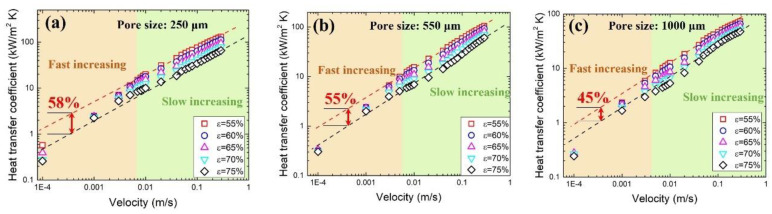
Variation in heat transfer coefficient with flow rate for samples with pore sizes of (**a**) 250 μm, (**b**) 550 μm, (**c**) 1000 μm and different porosities.

The heat transfer performance of metal foam is affected by the structure and flow behavior inside the foam. Figure 13 shows the variation in the heat transfer coefficient with velocity for samples with different pore sizes and porosities. Obviously, the heat transfer coefficient increases exponentially with flow rate. A fast-increasing regime and a slow-increasing regime can be identified over the entire range of flow rates. The fast-increasing regime is mainly in the low flow rate range where heat convection dominates the heat transfer. In the slow-increasing regime where the flow is moving fast, conduction dominates the heat transfer [14]. Figure 13 also shows that low porosity generally has a higher heat transfer coefficient. A red upper limit line and a black lower limit line are plotted in the figure. For a pore size of 250 μm, 550 μm and 1000 μm, the maximum heat transfer difference is 58%, 55% and 45%, respectively.

**Figure 14:**
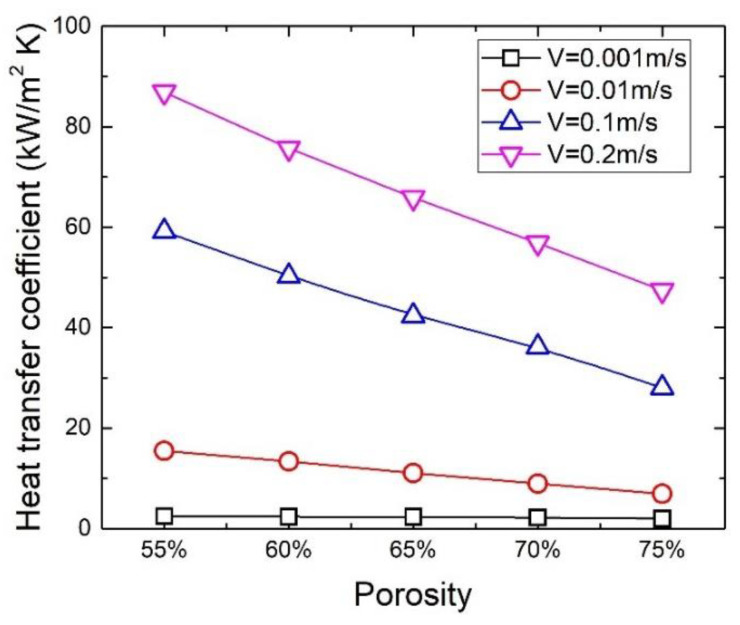
Variation in heat transfer coefficient with porosity for metal foams at different velocities.

Figure 14 plots the heat transfer coefficient as a function of porosity at different velocities. When the velocity is equal to or greater than 0.003 m/s, the heat transfer coefficient decreases with the increase in porosity because the thermal conductivity decreases with the increase in porosity. However, the effect of porosity on heat transfer disappears when the fluid moves at a velocity less than 0.003 m/s, which can be seen from Figure 13 and Figure 14. This is because at low velocity, the heat transferred by conduction through the metal foam is much greater than the heat removed by convection. At extremely low velocity, the intensity of convection is so weak that heat transfer between the metal foam and the fluid, as well as within the fluid, can be considered by conduction. Therefore, the effect of porosity on heat transfer is negligible.

**Figure 15:**
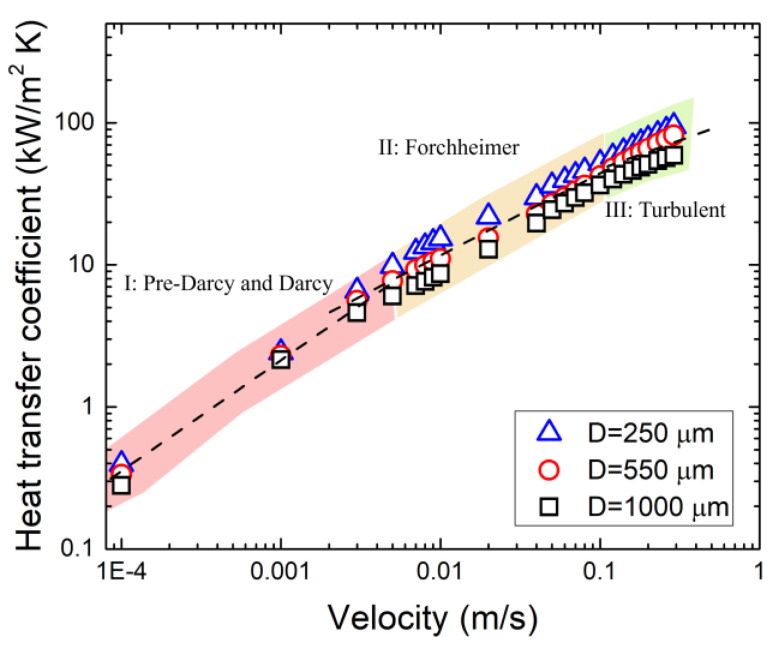
Variation in heat transfer coefficient with pore size for metal foam with different pore sizes.

Figure 15 shows the effect of pore size on heat transfer coefficients. Foam with a pore size of 250 μm showed the best heat transfer performance, followed by 550 μm and 1000 μm. Similar to the effect of porosity on heat transfer, the effect of pore size is also insignificant at extremely low velocity. In addition, the calculated results are consistent with our previous experimental results, in which the relationship between the heat transfer coefficient and velocity can be divided into three sections, as shown in Figure 15 [14]. As discussed in this section, the changes in the relationship are mainly due to changes in fluid flow behavior.

## 4. Conclusions

This paper numerically investigated the fluid flow behavior and heat transfer performance of open-cell copper foam by using ANSYS Fluent. A novel geometry model with varying porosity (55–75%) and pore size (250 μm, 550 μm and 1000 μm) was created based on the face-centered cubic arrangement of spheres. The flow velocity ranges from 0.0001 m/s to 0.3 m/s, covering pre-Darcy, Darcy, Forchheimer, and turbulent regimes.

(1) The effect of the number of model layers on the results is insignificant when the number of layers is equal to or greater than two. For the single-layer model, wall effects cannot be ignored, especially at high flow velocity.
(2) The pressure drop increased with decreasing porosity and increasing pore size. The relationship between the pressure drop and flow velocity can be correlated based on the Forchheimer equation. Permeability increased and the form drag coefficient decreased with increasing porosity and both increased with increasing pore size, but the pore size effect on the form drag coefficient is stronger.
(3) The laminar, transitional, and turbulent regimes were identified from the friction factor–velocity diagram. Low porosity and a small pore size results in a large friction factor. Flow in a large-pore-size model tends to become turbulent at a relatively low velocity.
(4) Temperature distribution is largely affected by flow velocity and porosity. Fast-moving fluid and high porosity results in an extremely uneven temperature distribution. Particularly, at high velocity, only a small thickness of metal foam close to the heat source contributes to heat transfer.
(5) Pore-scale velocity is hardly affected by porosity and pore size but is affected by flow rate. At high flow rates, vortices can be found in every pore.
(6) The heat transfer coefficient increased exponentially with flow velocity and increased with decreasing pore size and porosity. However, at extremely low velocity (≤0.001 m/s), the effect of porosity and pore size on heat transfer is negligible.
(7) The numerical results provide useful guidance for the design of metal foam heat exchangers. For example, increasing the thickness of porous media to a certain point no longer increases heat dissipation efficiency when the flow rate is high enough.

## Figures and Tables

**Table 1:** Diameter of the cylindrical hole and neighboring pore distance for all physical models.

Porosity	55%		60%		65%		70%		75%	
	*l_p_*	*r_c_*	*l_p_*	*r_c_*	*l_p_*	*r_c_*	*l_p_*	*r_c_*	*l_p_*	*r_c_*
250 μm	26.0	32.0	18.2	34.7	11.1	37.6	4.7	40.7	1.1	44.2
550 μm	57.3	50.2	39.9	55.0	24.4	60.3	10.4	66.4	2.3	73.5
1000 μm	104.2	69.3	72.6	76.2	44.4	84.1	18.9	93.3	42.6	104.3

**Table 2:** Boundary condition settings.

Boundary Name	Boundary Condition
Top surface	Constant heat flux
Front surface	Symmetric
Back surface	Symmetric
Bottom surface	Zero heat flux

**Table 3:** Permeability (*K*) and inertial drag coefficient (*C*) of all models.

	Pore Size					
	250 μm		550 μm		1000 μm	
Porosity	*K* × 10^−7^ (m^2^)	*C* (m^−1^)	*K* × 10^−7^ (m^2^)	*C* (m^−1^)	*K* × 10^−7^ (m^2^)	*C* (m^−1^)
55%	0.38	197.3	0.25	816.6	0.19	3000.0
60%	0.56	124.5	0.44	499.3	0.38	2000.0
65%	0.83	78.2	0.74	308.2	0.76	1000.0
70%	1.16	49.9	1.14	198.7	1.83	663.5
75%	1.49	32.1	1.82	117.9	2.75	397.9

## Data Availability

The raw data supporting the conclusions of this article will be made available by the authors on request.
